# Dietary Fibres in Processed Meat: A Review on Nutritional Enhancement, Technological Effects, Sensory Implications and Consumer Perception

**DOI:** 10.3390/foods14091459

**Published:** 2025-04-23

**Authors:** Marius-Mihai Ciobanu, Diana-Remina Manoliu, Mihai Cătălin Ciobotaru, Elena-Iuliana Flocea, Paul-Corneliu Boișteanu

**Affiliations:** 1 Department of Food Technology, “Ion Ionescu de la Brad” Iasi University of Life Sciences, 3 Mihail, Sadoveanu Alley, 700490 Iasi, Romania; marius.ciobanu@iuls.ro; 2 Faculty of Food and Animal Sciences, “Ion Ionescu de la Brad” Iasi University of Life Sciences, 8 Mihail, Sadoveanu Alley, 700489 Iasi, Romania; floceaiuliana5@gmail.com (E.-I.F.); paul.boisteanu@iuls.ro (P.-C.B.)

**Keywords:** functional food, dietary fibre, natural additives, nutritional enhancement

## Abstract

Meat is an essential source of nutrients in the human diet and a component of global food security. In the context of a growing demand for functional and healthy foods, the addition of non-meat ingredients, such as dietary fibres, is a promising strategy for improving the quality of meat products. This review aimed to identify and synthesise the available recent literature regarding the impact of fibre-rich ingredients on the properties of meat products, investigating how various plant sources (such as cereals, vegetables, legumes, and fruits) can be used in various forms of meat products, such as meat pastes, emulsified products, and minced and restructured meat products. Analyses of technological parameters revealed improvements in water-holding capacity, cooking losses, and an increased production yield. The addition of fibre has demonstrated a favourable effect on low-fat products, stabilising the emulsion and improving its physical texture properties. The chemical analysis highlighted an increase in dietary fibre and mineral content, as well as a decrease in fat content depending on the type and level of fibre added. Sensory changes included aspects related to the colour, aroma, texture, and overall acceptability of the products. The optimisation of the type and level of fibre is essential to obtain meat products with improved characteristics.

## 1. Introduction

Ensuring food security sustainably is one of the most pressing challenges facing humanity today and in the coming decades. Food security refers to the availability of food to meet nutritional and physiological needs, ensuring population well-being. Under these conditions, meat plays an essential role, being an important source of nutritional intake for many people around the world. With the rapid population growth and the simultaneous increase in living standards, the global demand for meat is continuously rising, with a tripling of global meat production over the past 50 years [1,2]. Global meat production exceeds 350 million tonnes each year, with a reported quantity of 371 million tonnes in 2023, 1.5% higher compared to 2022. The production increase was recorded for the meat from all species, but it is mainly due to the rise in poultry meat production, followed by pork, and finally beef and sheep meat [3]. Regarding global meat consumption, data reported by the OECD (Organisation for Economic Co-operation and Development) for the year 2024 show that the most consumed meat is chicken, with 22.1 kg per capita, followed by pork, with 20.8 kg per capita, beef, 13.7 kg per capita, and lastly sheep meat, with an average consumption of 1.1 kg per capita [4].

The increase in meat production and consumption is due, in addition to the need to provide food for the ever-growing population, to its high nutritional and biological value, especially as a good source of complete proteins, with meat traditionally being associated with good health and prosperity. Moreover, meat and meat products are ideal sources of soluble minerals, vitamins, essential amino acids, and other nutrients that have specific functions for the body [5,6,7,8]. In addition to the benefits and advantages brought by meat consumption, some negative aspects related to human health (mainly the high content of cholesterol and saturated fatty acids), animal health and welfare, and greenhouse gas emissions resulting from animal husbandry have been raised [9,10,11]. However, it is important to note that the negative aspects related to human health have mainly appeared in industrialised countries, where meat consumption often exceeded the recommendations of health and nutrition organisations [9]. At the same time, the impact of animal husbandry on environmental pollution is viewed differently depending on the context. In poor and low- to middle-income countries, livestock represent an important source of essential nutrients in the diet and satisfy certain culinary preferences, conferring a certain socio-economic status. In contrast, in high-income countries, the significant increase in demand and consumption of meat, due to rising purchasing power, along with the growing consumer awareness regarding ethical, animal welfare, and environmental issues, has exerted negative pressures on livestock farmers [11,12,13].

The changes in consumer demand, their higher acceptability for novelty, and the increased attention given to health-beneficial foods, as well as the need to improve the quality and negative image of meat, have prompted processors to find solutions by developing high-quality products with health benefits. In the meat processing industry, the incorporation of non-meat ingredients is carried out differently, taking into account the type of product. In minced meat products, the ingredients are added directly to the minced meat mixture, while in products with a compact meat structure, they are added through marination (immersion), injection, or vacuum tumbling [14]. The addition of non-meat ingredients in general, and, in particular, dietary fibres, has emerged as a solution to several issues, the most important of which are related to the following: (1) enriching the nutritional quality of meat products by adding elements that are not well represented in meat naturally, namely carbohydrate compounds (soluble and insoluble dietary fibres), to ensure a balanced diet with sufficient dietary fibre intake [15,16]; (2) the superior valorisation of certain plant by-products with the aim of reintegrating them into the valorisation stream to reduce waste in the plant processing industry [17]; (3) improving the technological and sensory properties of meat products [18,19].

The importance of fibre in the diet derives from its effect on intestinal motility and the prevention of constipation; it can facilitate weight loss by inducing satiety and reducing the frequency of food consumption. Fibre can regulate blood sugar levels, and studies are also examining the association between regular fibre consumption and the reduced risk of cardiovascular diseases [20], type 2 diabetes, and certain types of cancer [21].

In this context, this paper started with the initial question, “What happens when high-fibre non-meat ingredients are added to meat products? What are the main changes that occur, and what are the advantages?” The purpose of this review is to identify and map the literature that has evaluated the inclusion of high-fibre ingredients in meat products.

## 2. Aspects Regarding the Necessity of Enriching Meat Products with Fibre Sources

Meat is recognised as an essential source of easily digestible proteins with high biological value due to its balanced content of essential amino acids [22], as well as lipids rich in linoleic acid, a polyunsaturated fatty acid that has been identified with a series of beneficial properties for promoting health [23,24].

Meat, as muscle tissue, is presented in specialised literature as being composed of approximately 72–75% water and 25–28% dry matter, consisting of 19–20% proteins and 1.5% non-protein nitrogenous compounds (which include nucleotides, peptides, creatine, and creatinine), 2.5–5% lipids, 1.2% carbohydrates, 0.65–1% ash, and 0.1% vitamins. Vitamins, including the B vitamin complex, and minerals, including valuable trace elements like iron, iodine, and zinc, along with useful amounts of copper, magnesium, cobalt, phosphorus, chromium, and nickel, play a significant role in the chemical composition of meat [5,25]. Certainly, there are variation factors for the content of macro (proteins, lipids) and micro (vitamins and minerals) nutrients in the product generally referred to as meat. The most frequent differentiations regarding the chemical composition of meat occur between the meat of different animal species and between the analysed muscle regions (Table 1), with lipids being the most variable component, as their values can vary widely between 1% and 15% [26,27].

However, carbohydrate compounds, which together with lipids and proteins make up the three main macronutrients, are present in living muscle tissue in relatively small concentrations, within the range of 0.5% to 1.5%. The main carbohydrate is glycogen, a branched-chain polysaccharide, which in the living animal is stored in the liver and serves to provide energy for muscle contraction through the process of aerobic glycolysis. After carcass processing, due to the unavailability of oxygen in the blood, an anaerobic process occurs in which glycogen is converted to glucose, that is then transformed into lactic acid. In the early stages of meat maturation, the glycogen concentration drops below 1% [26,43,44].

In the composition of meat, within the carbohydrate category, in addition to glycogen, there are glucose, other mono- and disaccharides (0.1–0.15%), as well as intermediates of glycogen metabolism [26]. The lack of indigestible polysaccharide compounds from the fibre class is a major disadvantage of meat, as contrary to the increasing consumption of meat, a healthy diet must be supplemented with plant-based products, recognised as excellent sources of fibre [45,46].

Dietary fibre (DF) consumption is well-recognised for its benefits to human health, particularly in supporting cardiovascular health, due to its ability to lower blood cholesterol and triglyceride levels [47]. It also contributes to gastrointestinal health by reducing transit time, diluting and binding toxic colon chemicals, thereby decreasing mucosal exposure to harmful substances [48]. Moreover, fibre consumption is associated with improved glycaemic regulation, effective weight management, and a reduced risk of chronic conditions, including type 2 diabetes and obesity [49].

According to the Dietary Reference Intakes (DRI), the recommended fibre intake, per 1000 kcal, is 14 g [50]. The recommended daily intake of dietary fibre for children and adolescents varies from 10 to 40 g, depending on factors such as age, gender, and energy consumption. Edwards et al. [51] state that the European Food Safety Authority (EFSA) recommendations for fibre intake in children are 10 g/day for ages 1–3 years, 14 g/day for ages 4–6 years, 16 g/day for ages 7–10 years, and 30 g/day (M)–40 g/day (F) for ages 14–18 years. While for adults, the recommended fibre intake is 30–35 g per day for men and 25–32 g per day for women [21] to maintain a healthy body, an intake primarily supported by the consumption of cereal products, vegetables, fruits, and oilseeds [45,52].

Carbohydrates, together with fats, represent the most important sources of dietary energy for the body. The energy contribution of carbohydrates varies depending on the class they belong to. Starch, a polysaccharide, is responsible for the largest amount of energy in global nutrition, followed by the monosaccharide’s glucose and fructose, as well as the disaccharides sucrose, maltose, and lactose [53]. Also classified within the carbohydrate category, fibres include those non-starch polysaccharides, their essence being represented by their indigestibility, which in turn is due to their molecular size, comprising over 10 monomers [21]. Dietary fibres are mainly derived from various edible components of plants that constitute part of the cell wall structure of fruits, vegetables, and whole grains [54]. These compounds are macronutrients with a low-calorie content, constituting a large part of the dry matter in many plant foods, and are associated with significant health benefits and a healthy lifestyle [22,53].

To meet the demand of consumers who pay increased attention to health, new opportunities are being explored in the meat industry for the production of functional meat products enriched with non-animal bioactive compounds, including dietary fibres from various sources [55]. In addition, meat and meat products are excellent options for introducing deficient nutrients into the diet due to their high level of acceptability and frequency of consumption, their contribution to the dietary intake of essential nutrients, and their high bioavailability [22,55].

Moreover, the lack of carbohydrate components associated with the lipid content specific to the type of meat can predispose meat products to more pronounced oxidative deterioration [22]. Thus, in addition to the nutritional contribution of fibres, the introduction of non-animal ingredients from the class of vegetable fibres also plays a role in slowing down the oxidative deterioration of meat products through their antioxidant properties [56,57,58,59,60].

## 3. Meat Products Suitable for Fibre Addition

Meat products, whether minced—with a heterogeneous structure—or compact—with a homogeneous structure, do not serve as sources of dietary fibre, despite the inclusion of spices that may contain a certain amount of fibre [61]. The interest in integrating dietary fibres into processed meat formulations arises from the enhancement of functional and sensory attributes, the improvement of nutritional content, and the necessity to satisfy consumer demands and preferences. This approach contributes to the advancement of the food industry, thus observing a noticeable increase in the use of dietary fibres in these formulations [22].

Incorporating a specific fibre into a meat product involves several factors, including the technological properties of fibre (such as concentration, particle size, water retention capacity, and swelling capacity), physicochemical properties (pH, antioxidant activity, pro-oxidant activity, composition, and type of fibre—soluble/insoluble), as well as sensory properties [61,62]. The attributes of non-meat ingredients, particularly dietary fibres, induce alterations in the product’s structure—technological, sensory, colour, and texture—that must be assessed and taken into account in the production process development.

Furthermore, the selection of fibre type must consider the product’s structure and the specific manufacturing processes involved [63]. Thus, based on the structural integrity of the meat utilised as raw material, meat products may display either a complete muscle structure or a heterogeneous structure. Products with a compact structure are composed of sections or whole pieces of meat, typically seasoned and subjected to thermal processing [64]. The phrase “minced meat” implies meat products, whether raw or cooked, that have been sliced, minced, or chopped into small fragments. The formulation of these ground meat products typically comprises a blend of meat and non-meat constituents, with the meat element frequently consisting of semi-lean meat, generally characterised by higher fat content [64,65].

Depending on the degree of grinding, minced meat products can also be divided into ground meat products or emulsified products [64]. These products with a disintegrated/ground structure, and especially the emulsified products, contain a relatively high amount of fat (20–35%), mainly represented by pork fat [66]. The role of fat in these products is to contribute to the stability of the meat emulsion, reduce cooking losses, and improve texture and juiciness during consumption [67]. In this context, the use of various fibres is evaluated in numerous studies to reduce fat content and improve the quality properties of different meat products, without negatively affecting the yield of the production process.

The efficiency of introducing different fibres into emulsified meat products (e.g., bologna sausages, frankfurter-type sausages, and nuggets) has been evaluated in several studies, for example, the addition of green banana flour [68,69], regenerated cellulose fibre [67], amorphous cellulose fibre [70], and rice bran fibre [71]. Regarding minced products with heterogeneous structure (such as sausages and salami) studies present the following fibres used: red dragon fruits [66], apple, orange, and peach fibres [72]; while for restructured meat products (meatloaf, restructured meat slices): flaxseed flour [73], apple, oat, pea, and inulin [74]; and for semi-prepared meat products (burgers, meat patties): oat soluble fibres [75], guava, and tomato [76].

Numerous studies present formulations of common minced meat products improved by the addition of various dietary fibres, their minced structure making it possible to incorporate the innovative element, most often in powder form (flour), as shown in Table 2.

## 4. Dietary Fibres: Classification, Categorisation, and Application in Meat Products

The class of dietary carbohydrates includes monosaccharides, disaccharides, oligosaccharides, and polysaccharides, with the latter being further divided into starch and non-starch polysaccharides. The general conception of dietary fibres is that they are, more or less, synonymous with indigestible or “unavailable” carbohydrates [104]. The category of indigestible carbohydrates includes non-starch polysaccharides (carbohydrate fractions, excluding starch and free sugars), resistant starch (RS), and oligosaccharides [105,106]. Resistant starch is also considered insoluble dietary fibre because it cannot be digested in the small intestine. However, this classification applies only when resistant starch is naturally present in foods. If derived through artificial synthesis (physical, enzymatic, or chemical processes), resistant starch must demonstrate desirable physiological benefits to be classified as dietary fibre [105,107]. Oligosaccharides, characterised as indigestible and resistant carbohydrates with degrees of polymerization between three and nine, are classified as dietary fibre by the Codex Alimentarius due to their similar physiological functions [105,106].

Polysaccharides constitute the primary components of plant cell walls [108]. Cellulose, a polysaccharide consisting of β-glucose, is the most widespread and abundant insoluble fibre in nature. It possesses a high molecular weight and is a significant component of plant cell wall structure, alongside hemicellulose, pectin, and lignin [106]. Hemicellulose consists of a branched chain of smaller β-glucose monomers that encompass various sugars, including xylose, mannose, and arabinose [109].

Pectin can be defined as a collection of heterogeneous polysaccharides, presenting a varied structure based on the plant’s botanical origin, and is extensively found in fruits and vegetables. This compound is regarded as one of the most intriguing cell wall polymers because of its structural, functional, and technological attributes, including its distribution in plants, water solubility, reactivity to chemical processes, and diverse industrial applications [108]. Lignin, a non-carbohydrate polymer, is a complex, high molecular weight structure composed of cross-linked phenolic monomer polymers. Lignin, in the primary wall of plant cells, offers structural support, impermeability, and resistance to microbial invasion, and is classified as dietary fibre [106,110].

Dietary fibres can be classified based on three main criteria: source, structure, and solubility [109]. Based on their source of origin, dietary fibres are categorised into plant-based fibres, synthetic fibres, animal-derived fibres, and microbial fibres [109,111,112]. Among these, plant-derived fibres are the most studied and are further divided into three subgroups: (i) natural carbohydrate polymers, which occur naturally in edible plants and are introduced into the human diet through the consumption of vegetables, fruits, seeds, cereals, legumes; (ii) edible carbohydrate polymers, which are extracted from raw foods through physical, enzymatic, or chemical processes and provide physiological benefits (e.g., resistant oligosaccharides, inulin, and psyllium); and (iii) synthetic carbohydrate polymers, which have been demonstrated to offer physiological benefits to the body (e.g., methylcellulose) [106].

From a structural perspective, dietary fibres can be classified as either linear polysaccharides or branched (nonlinear) polysaccharides. However, the most widely accepted classification system for dietary fibres is based on their water solubility and fermentative behaviour when subjected to enzymatic action simulating the human digestive process in vitro. According to this criterion, dietary fibres are categorised into insoluble dietary fibres (IDF) and soluble dietary fibres (SDF). IDF, which includes cellulose, portions of hemicellulose, and lignin, undergoes minimal fermentation. In contrast, SDF consists of well-fermented fibres such as non-cellulosic polysaccharides, oligosaccharides, and certain indigestible polysaccharides, including inulin, gums, pectins, galactomannan, and β-glucans [109,113].

The intake of dietary fibre is primarily ensured through the consumption of plant-based foods, including vegetables, grains, woody plants, fruits, nuts, and legumes. Dietary fibres and functional food products have gained importance due to their nutritional significance and high market potential [46]. Additionally, incorporating fibre as an ingredient in the food industry adds value by enabling the efficient utilisation of by-products from fruit and vegetable processing—such as peels, seeds, and stems—aligning with current trends toward sustainability and the transition to a circular economy [114].

When used as an ingredient, dietary fibres must meet specific criteria: a total fibre content of more than 50%, low water and lipid content, low caloric value, and a neutral flavour [18,46]. Moreover, the incorporation of dietary fibre as a functional ingredient in food products requires consideration of its technological properties, including hydration capacity (swelling ability, gel-forming ability, water retention, and absorption), solubility, viscosity, and its ability to adsorb and bind ions and organic molecules [115]. Those functional properties of fibres extracted from various plant sources influence the quality and characteristics of meat products [116,117].

Dietary fibre such as dehydrated fruit, vegetable, and cereal fibres (Table 3) are an appropriate functional ingredient for the preparation of meat products due to their water retention properties, which help reduce cooking loss and maintain moisture levels, yielding excellent outcomes [99,108,118,119]. Moreover, as they have the ability to enhance water retention, their incorporation into a meat mixture helps to preserve its juiciness. Consequently, this results in a slower release of the volatile compounds responsible for the product’s flavour [46].

Identifying the optimal source for increasing fibre intake and achieving the desired technological properties of additives in meat products requires an understanding of fibre’s moisture retention and oil-binding capacities. These properties are influenced by the botanical source, fibre content, the ratio of insoluble to soluble fibres, and particle size [127].

Zhao et al. [128] reported that reducing the particle size of rice bran through superfine processing to obtain an insoluble fibre powder increased its water-holding capacity (WHC) and swelling capacity while decreasing its oil-holding capacity. Moreover, superfine grinding has been shown to enhance the functional properties of insoluble dietary fibre derived from rice bran, including the extractability of free and bound phenols, phenolic bioaccessibility, and antioxidant activity, making it more suitable for application in functional foods.

Similarly, in a study conducted by Ma and Mu [129], fibres obtained from defatted cumin through different extraction methods demonstrated improved functional properties with increasing sieve mesh size. The optimal physicochemical properties were observed within a range of 40–120 sieve mesh size. The behaviour of added fibres in the meat product matrix is directly influenced by their initial functional properties. Table 4 provides a comparative overview of the key technological characteristics of some dietary fibre sources, including water and oil-holding capacity, gel-forming ability, and swelling capacity.

## 5. Influence of Dietary Fibre Addition in Meat Products

When integrated into meat formulations, dietary fibres influence the quality of the product through their effects on nutritional, physicochemical, technological, and sensory properties [22,61,141].

### 5.1. The Effect on Technological Properties

The technological properties of processed meat products are key parameters in determining quality and significantly impact consumer acceptance [142]. Cooking yield, emulsion stability, and water-holding capacity are technological attributes that influence both production costs and the development of desirable quality characteristics in processed meat products. Dietary fibres are generally associated with a high capacity for water retention and binding, as well as a strong oil-binding capacity. However, these technological properties are influenced by both source and quantity of fibres incorporated into the formulation [143].

#### 5.1.1. Influence on the Acidity of Meat Products

The acidity of processed meat products, most commonly expressed as pH, influences their safety, sensory acceptability, technological characteristics, and storage stability [144,145]. pH variations may occur depending on the initial pH of the applied addition. In this context, the enrichment of poultry roast with hemp seeds, flour, and oil does not cause significant changes in the final product’s acidity, regardless of the quantity introduced [145]. The replacement of sodium nitrite with white kimchi powder (0.2% and 0.4%) and acerola juice powder (0.1% and 0.2%) determined a decrease in the pH of processed meat products due to their naturally more acidic pH [146]. This effect was more pronounced in formulations with a higher percentage of acerola juice powder due to its inherently lower initial pH (3.33).

In the development of phosphate-free frankfurter sausages, Yuan et al. [83] incorporated soluble fibres from marine algae in five concentrations (0.25%, 0.5%, 0.75%, 1.00%, and 1.25%). Given the alkalising effect of phosphates, the study reported lower pH values in phosphate-free sausage samples. Moreover, as the percentage of marine algae fibres increased, product acidity decreased, with higher concentrations (1.00% and 1.25%) significantly improved pH levels—an effect attributed primarily to the weak alkalinity of the marine algae additive. Similar findings were reported by Tomar et al. [147], who observed an increase in the pH of some ready-to-cook chicken cutlet formulations as the proportion of dehydrated vegetables increased, likely due to the alkaline nature of the vegetable mix.

Madane et al. [148] conducted another notable study on the efficacy of dragon fruit peel powder as an antioxidant dietary fibre (1.5% and 3%) in chicken nuggets. The study reported a significant decrease in the pH of chicken meat emulsion following the incorporation of dragon fruit peel powder, with values declining from 6.37 in the control sample (without addition) to 6.31 for the 1.5% formulation and 6.18 for the 3.0% formulation. The authors attributed this pH reduction to the inherently acidic nature of dragon fruit peel powder (pH 5.54) and observed a positive correlation between the extent of pH decrease and the percentage of the additive used.

#### 5.1.2. Influence on Water-Holding Capacity

Water-holding capacity is an important characteristic of meat products, as it directly influences their sensory attributes. In their study, Madane et al. [148] reported that the incorporation of dietary fibres from dragon fruit peel enhanced water retention capacity by modifying texture, improving moisture retention, and reducing cooking losses compared to the control sample. Araujo-Chapa et al. [149] observed a similar trend, demonstrating that the addition of soy husk dietary fibres in frankfurter sausages (1% and 1.5%) increased water retention capacity, with a more pronounced effect at higher fibre concentrations.

Xu et al. [150] studied the mechanism of improving the water retention capacity of meat paste by adding insoluble fibres from anise in proportions of 1%, 2%, and 3%. The authors studied water retention capacity and cooking losses over a 7-day storage period. The three formulations with insoluble anise fibres showed lower cooking losses compared to the control sample, explained by the fact that the hydrophilic groups of the insoluble fibres absorb water and help fill the protein network through inactive effects. Moreover, the increase in cooking losses with increased storage time was not as intense, especially for the formulations with 2% and 3% anise fibres. Correlating the moisture content of the samples with cooking losses, the authors found that the improvement in water retention capacity was due to the effect of anise fibres and not to differences in the moisture content of the formulations. Thus, the addition of anise insoluble fibres cannot prevent the decrease in the water retention capacity of the emulsified paste with increased storage time; however, it could delay the increase in losses during thermal treatment.

Biswas et al. [80] highlighted similar results in their study of the effect of dragon fruit peel powder into fish nuggets (1.0%, 1.5%, and 2.0%). The addition of dragon fruit peel powder significantly improved both the emulsion stability and the cooking yield of fish nuggets, with the presence of dietary fibres enhancing water retention capacity.

#### 5.1.3. Influence on Cooking Yield

Authors reported that incorporating a dehydrated vegetable mix (2.5%, 5%, and 7.5%) into ready-to-cook chicken cutlet formulations [147], respectively, hemp-derived ingredients (seeds/flour/oil) into two poultry roast formulations (8/0.2/2% and 4/0.2/6%) [145] resulted in a significant increase in product yield, with the increase being directly proportional to the amount of added mix, and also lower thermal processing losses, compared to the control sample. Notably, formulations with a higher hemp seed content (8%) exhibited reduced thermal losses, an effect attributed to the fibre content of the seeds and flour.

Das et al. [25] demonstrated that the inclusion of moringa pod powder in goat nugget formulations significantly improved cooking yield, with no significant differences observed between the two tested concentrations (1.5% and 3%). The authors attributed this effect to the increased surface area and porosity of moringa pod fibre, which facilitated greater water and fat retention, thereby minimising cooking losses and enhancing yield. Conversely, Kim and Shand [86] reported that the addition of lentil flour and beetroot powder did not significantly impact cooking losses in pork bologna sausages, irrespective of the percentage introduced (6% for lentil flour and 0.1%, 0.3%, and 0.5% for beetroot powder). The authors state that the lack of effects on cooking losses can be explained by the use of water-impermeable membranes, which prevent excessive water loss during cooking. Moreover, although lentil flour increased the viscosity of the raw batter and reduced expressible moisture and purge loss, its effect on cooking losses was not significant, as the product already retained water efficiently. Another possible factor is the partial gelatinization of starch in lentil flour, which influenced the structural properties of the mixture without affecting cooking losses.

The introduction of dietary fibres as a fat replacer can influence the effect of increased cooking yield typically associated with fibre addition. Corimayhua-Silva et al. [66] investigated this phenomenon by substituting pork fat with hydrated dragon fruit peel powder in emulsified sausages at levels of 3.29%, 6.57%, and 9.86%. The study reported a negative correlation between the percentage of pork fat replacement and cooking yield (72.2–66.9%) and a positive correlation with frying losses (27.8–33.0%).

Similar findings were reported by Jovanovichs et al. [151] in sausage formulations, where pork back fat was replaced either with a combination of flaxseed oil and psyllium fibre gel (15% and 30%) or solely with psyllium fibre gel (15% and 30%). The highest weight losses after thermal treatment were observed in formulations containing only fibre gel, particularly at the highest level of fat substitution (30%). These results can be attributed to the lower water content of fat compared to lean meat, and reducing the fat content while maintaining the same level of fibre leads to increased water losses, ultimately resulting in a lower cooking yield [151,152].

#### 5.1.4. Influence on Emulsion Stability

Another indicator of the technological quality of processed products, specific to emulsified meat products, refers to emulsion stability [83]. The viscosity of meat paste, which reflects emulsion stability, correlates with its water- and fat-retention capacity [86,153]. Studies have shown that emulsion stability is improved in products with added fibres, such as buckwheat powder [153], dragon fruit peel [148], cellulose fibres [67], beet powder, lentil flour [86], and inulin-based emulsion gels [123].

Schmiele et al. [70] studied the use of amorphous cellulose fibres (0% for the control sample, 0.2%, 0.75%, 1.3%, and 1.5%) for fat replacement in emulsified meat products. Optimal results regarding emulsion stability, similar to a standard sample, were obtained for the formulation with 1.3% cellulose fibres and a fat replacement degree of 50%. The authors stated that the addition of amorphous cellulose fibres and pork fat significantly increased emulsion stability, as well as consistency and hardness. Another experiment evaluated the use of buckwheat powder (1%, 2%, and 3%) in meat emulsion for sausage production [153]. The applied addition led to changes in viscosity due to increased water retention promoted by the fibres contained in the buckwheat powder, and the increase in viscosity led to higher stability of the meat emulsion, which was directly proportional to the percentage of powder added.

#### 5.1.5. Influence on Diameter Reduction

**Figure 1:**
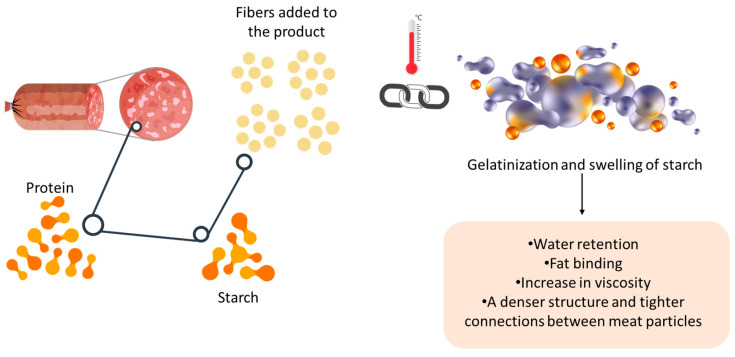
Schematic representation of the effect of the interaction between meat proteins and the fibres and starch in vegetable powders (especially those rich in starch, such as legumes) during heat treatment.

In the case of meat burgers or meatballs, a technological characteristic related to diameter reduction or shrinkage appears, which occurs due to weight loss, protein denaturation and contraction, water evaporation, and fat drainage from the product [103]. Fibres can form a gel that contributes to increased viscosity [154], while the presence of starch and proteins facilitate water retention and fat binding in the product during thermal treatment through the phenomenon of protein gelatinisation and starch swelling (Figure 1) [103,155]. Thus, the addition of plant fibres can minimise the effect of diameter shrinkage by better retaining water and fats [117,126].

In the case of hybrid chicken burger formulations, the addition of legumes (peas, chickpeas, and lentils) significantly decreased diameter reduction and thickness reduction compared to the control sample; however, at a 75% addition diameter reduction slightly increased, while still remaining significantly lower than the control [103]. Argel et al. [126] also reported that the incorporation of legume flour (pea, chickpea, lentil, and bean) led to a reduction in diameter shrinkage after thermal treatment for all burger formulations (1%, 2%, 3%, 4%, 5%, and 6%) compared to the commercial control product. This suggests that at higher inclusion levels, the effect of improving cooking yield and diameter reduction reaches a maximum, after which it registers a decline. Moreover, with the improvement in yields and diameter reduction during cooking, and with better retention of oil and water in processed meat products, there is a decrease in expressible fluid [86,126].

#### 5.1.6. Influence on Water Activity

Water activity in meat products is a relevant factor regarding product stability during storage, as it allows the occurrence of various microbiological and biochemical processes [144]. Water activity is closely related to the amount of water in the product and implicitly to the amount of fat contained. Momchilova et al. [156] studied the effect of replacing pork back fat with inulin gel and oat bran flour in formulations of meatloaf made from beef, turkey, and poultry liver. They reported an increase in water activity, which was more significant in the formulation with complete fat replacement with equal amounts of inulin gel and oat bran flour (185 g·kg^−1^/185 g·kg^−1^).

Moreover, the increase in water activity reported by Kerner et al. [144] in samples of grilled meatballs enriched with mechanically pressed and dried hemp seed cake and sweet grass extract in two proportions (2%/0.5% and 1.5%/0.5%) was maintained even during the 21-day storage period. Between samples with different formulations of the applied additive, the authors did not report significant differences in water activity; however, the applied statistical test highlighted significant differences in the formulations compared to the control sample.

### 5.2. The Effect on Physical Quality Attributes

The incorporation of dietary fibres into meat products has increased in popularity due to health benefits associated with these compounds. Similarly to the changes that occur in technological quality of meat products with the addition of dietary fibres, the physical characteristics, such as texture and colour, undergo specific modifications depending on the added fibres.

#### 5.2.1. Influence on Meat Product Texture

Texture, appearance, and taste are the three main factors influencing food acceptability. Although human perception of texture cannot be entirely replicated, instrumental measurements play a key role in its analysis. The most commonly used instruments for assessing texture include the Instron, Warner-Bratzler press, Kramer Shear device, Brookfield CT3 texture analyser, and Lloyd LS5 [62,108,157].

Texture is a critical determinant of both quality and consumer acceptability of meat products. Consumers have specific expectations regarding attributes such as firmness, juiciness, and chewiness. Modifying texture through the incorporation of dietary fibres can influence consumer perception, acceptability, and purchasing decisions. Studies have shown that the addition of dietary fibres to meat products generally leads to significant textural changes, often increasing firmness. Kaur et al. [158] demonstrate these effects by incorporating powder from cauliflower stems and leaves into meat cutlets, an addition that significantly increased hardness, elasticity, cohesiveness, chewiness, and gumminess compared to control sample, an increase directly correlated with the level of powder added. This effect is primarily attributed to the reduction in fat content, which promotes the formation of a denser structure and stronger interparticle connections, thereby enhancing hardness, cohesiveness, and chewiness [62].

The type of fibre applied also holds particular importance in textural changes. Kim and Shand [86] state that the hardness of some bologna sausage samples incorporated with lentil powder was significantly higher compared to that of the control batch, while the incorporation of beet powder at levels of 0.1%, 0.3%, and 0.5% did not lead to significant differences in texture properties. Another study that showed an improvement in turkey pâtés texture was the addition of rice, oats, corn, and buckwheat [86]. This study found that the hardness, elasticity, and chewiness of pâtés with plant-based supplement increased moderately, while gumminess and cohesiveness remained similar to those of the control group. These texture improvements are consistent with findings from other studies, such as the reduction in hardness (from 49.9 to 34.8 N) and chewiness (from 26.9 to 21.7 N) of alpaca meat sausages formulated with red dragon fruit peel [66].

Salejda et al. [84] suggest that adding buckwheat husks modify the texture profile, more noticeably after two weeks of storage. Even though on the day of manufacturing sausages with 1%, 2%, and 3% buckwheat bran, the hardness decreased, and elasticity, gumminess, chewiness, and cohesiveness did not change significantly, the differences that appeared after two weeks were more evident. Therefore, after this period, the products were more compact and firmer compared to the control samples without buckwheat husk. The increase in hardness could be explained by the ability of plant fibres to form a stronger three-dimensional network within the meat matrix.

At higher incorporation percentages (5% and 7%) of insoluble kiwi fibre, the texture of pork meatballs changed significantly. The results reported by Zhao et al. [97] showed that hardness initially decreased significantly with the increase in the content of insoluble kiwi fibres, followed by a sharp increase from 5% addition level, with the group containing 7% insoluble kiwi fibres exhibiting the highest hardness (34.57 N/cm^2^). This biphasic response could be explained by two concurrent effects: at low levels of insoluble kiwi fibres, the water retention capacity of fibres increase the moisture content of the meatballs, and as the fibre content further increases, the small particles of insoluble fibres can fill the gaps in the protein network, leading to increased hardness. Similarly, chewiness and gumminess also increased significantly compared to the control batch, due to the gel-like properties of kiwi fibres, which contribute to a strong viscosity [97].

#### 5.2.2. Influence on Meat Product Colour

Colour is the consumer’s first contact with the product, and it largely determines the consumer’s initial perception of the quality of meat products. The colour characteristics of cooked meat products mainly derive from the meat’s initial pigmentation and the additives used in formulations [89]. Therefore, the ingredients added to meat products during the manufacturing process contribute to colour changes, which are influenced by their initial degree of pigmentation.

The addition of legume flour significantly affected the colour of burgers compared to the control group [126]. Lentil flour, at two levels of addition (80 and 150 g kg^−1^), resulted in the lowest L* values, while bean flour produced the highest brightness for the burgers (the highest L* values). Pea, chickpea, and broad bean flours all exhibited a greener hue (lower a* values) compared to the control, while lentil flour had the opposite effect, resulting in higher a* values (a more intense red colour), likely due to its higher carotenoid content. These lentil flour burgers even resembled the commercially available pork product in terms of the red hue intensity. All burgers formulated with pea and chickpea flour exhibited higher b* values compared to the control group. In contrast, lentil flour (except for the lower level) and all additions of bean flour resulted in burgers with a less yellow hue (lower b* values). Similar results were obtained by Boișteanu et al. [101] where the addition of red lentil flour (5% and 10%) in beef burgers reduced brightness and increased the intensity of the red colour (a*).

In another study, Choe et al. [159] investigated the effect of winter mushroom powder on the colour of emulsified sausages as a potential substitute for phosphates. Sausages with more than 1.0% winter mushroom exhibited significantly higher (a*) values than the phosphate control, while lightness (L*) values remained similar. Thus, the authors concluded that the applied addition is less likely to cause discolouration issues, even though the colour of winter mushrooms is white, making their use as a natural additive in meat products advantageous. In contrast, Reddy et al. [73] reported a significant decrease in lightness (L*) and redness (a*) with the addition of flaxseed flour in restructured buffalo meat slices, while b* values were not significantly affected by this addition.

### 5.3. The Effect on the Chemical Composition

**Figure 2:**
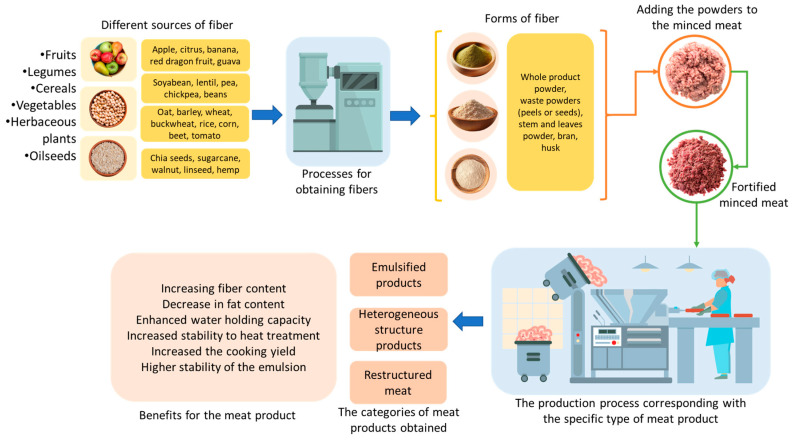
Schematic diagram for the enrichment of meat products with dietary fibres: sources of fibres, forms of addition (predominantly powders), compatible categories of meat products, and the functional benefits of this additions.

The nutritional properties of meat mainly depend on its chemical composition, which varies according to numerous extrinsic factors such as anatomical position of the muscle, genotype and age of the animal, and the quality of the feed. All these factors have a significant impact on the quality of processed meat products. The addition of health-promoting ingredients, such as dietary fibres, can contribute to improving nutritional profile of processed meat products and the healthy appearance of meat products [160]. However, the behaviour of the product after the addition of fibres from different sources (grains, fruits, vegetables, legumes, and oilseeds) varies depending on the type introduced and its proportional level in the product (Figure 2).

#### 5.3.1. The Impact of Cereal-Based Fibres

Cereals such as wheat, rye, and rice are considered good sources of insoluble dietary fibre, while oats, in the form of flour or bran, are a recognised source of soluble fibre. Due to their high water retention capacity, adding them as ingredients in low-fat meat products helps maintain moisture [143].

In an attempt to replace fat with inulin gel and oat bran flour in a range of emulsified meat products (Leberkäse), Momchilova et al. [156] demonstrated that the use of functional additives had a significant effect on the chemical composition of the finished products. Replacing fat with inulin gel and/or oat bran flour resulted in products with reduced energy value, which was inversely proportional to the percentage of fat replacement. The reduction in fat was maximal in the sample with complete fat replacement with oat bran flour, followed by the sample formulated with a combination of pork fat, inulin gel, and oat bran flour. The highest water content was recorded in the sample formulated with equal amounts of inulin gel and oat bran flour. The water percentage was higher in the samples with oat bran flour, followed by the samples with inulin gel, due to better water retention and the hygroscopic properties of oat bran flour. Additionally, inulin caused a more pronounced increase in carbohydrate content, and implicitly in fibre, compared to the addition of oat bran flour.

Similar results were obtained by Ferjančič et al. [87], who reported a decrease in fat content in chicken bologna sausages following the formulation of recipes with added inulin, oats, or psyllium (3% and 6%). Additionally, the most significant increase in total dietary fibre was observed with psyllium fibre enrichment, with the 3% formulation achieving 7.4% fibre and the 6% formulation achieving 10.57% fibre. The authors attribute these values to the dietary fibre content in the psyllium addition, noting that while the specified fibre content in psyllium was 85 g/100 g, their results indicated a higher content of 95.88 g/100 g.

Słowinski et al. [161] studied the possibility of producing sensory and technologically acceptable canned meat containing 6% wheat fibres to be labelled as “high-fibre content” products. In this study, the influence of the dose and length of wheat fibres on the quality of sterilised meat cans was investigated. The authors highlighted that the addition of wheat fibre preparations, with fibres of different lengths, in proportions of 3% and 6% did not affect the basic chemical composition (water, protein, fat, collagen, and salt content), water activity, and pH of the sterilised meat.

#### 5.3.2. The Effect of Vegetable-Based Fibres

Legumes contain significant amounts of starch, fibre, and protein. Starch enhances the development of heat-induced robust structures by swelling the granules in the protein gel matrix, thereby increasing its ability to bind water [126]. Pea fractions, such as starch and fibres, have been used as functional components in ground meat products, serving as texture-modifying or binding agents through their protein-binding properties [155]. Following the applied additions, the authors observed insignificant differences in the chemical constituents between treatments for raw burgers, while, after cooking, a significant increase in fat content was observed with the increased proportion of peas, while protein and moisture content remained constant.

Lentils have also been studied as a functional ingredient in meat products, alone or in combination with beets, chickpeas, and beans [86,162]. In general, the addition of lentil flour results in a decrease in product moisture, along with increases in protein and lipid content.

A similar study was conducted by Chandler and McSweeney [103], who determined the nutritional quality of hybrid burgers formulated with poultry meat and legume flour (yellow pea, chickpea, and lentil, in proportions of 25%, 50%, and 75%). The moisture content of the products after cooking decreased with the increase in legume content. The most significant decrease in moisture was observed in products with added yellow peas, from 61.27% in the control sample to values of 58.66% with a 25% addition, 52.54% with a 50% addition, and 48.96% with a 75% replacement. The control burgers and those with 25% had a significantly higher moisture content in contrast to those with 50% and 75%, a trend that the authors attributed to the fact that legumes have a lower moisture content than chicken meat.

In addition, the level of pulse flour positively correlated with the fat content of the cooked products, except for the 75% formulations, in which the fat content decreased compared to the previous formulation, while still exceeding the control group. This result can be attributed to the initially lower fat content of the 75% formulation. Compared to the control burger, which had a significantly lower fat content, all three burgers with 50% legumes (yellow pea, chickpea, and lentil) showed higher fat levels (*p* < 0.05) after cooking [103]. Fat plays an important role in burger quality, contributing to flavour retention and enhancing the perception of juiciness and tenderness [163].

Similar results were obtained by Shoaib et al. [164], reporting a directly proportional increase in fat content following the inclusion of pea protein isolate at concentrations of 3%, 6%, 9%, and 12% in chicken nugget formulations. In contrast, the addition of rice protein isolate did not follow the same trend of increasing fat content, with results showing a decrease in lipid levels at a 3% addition (7.93% fat, compared to 8.14% fat in the control sample), followed by a significant increase at a 6% addition of rice protein isolate (9.60% lipids).

In a similar manner, the use of pea protein isolates in chicken nuggets led to an increase in fat content as the amount of pea protein isolates increased [164]. In contrast to these findings, raw burgers exhibited a decrease in fat content following the addition of legumes. This phenomenon can be explained by the tendency of plant proteins to retain fat in food products, as fat binds to plant proteins during thermal processing [103].

Contrary results were reported by Banerjee et al. [77], who investigated the impact of enoki mushroom stem by-products on the quality characteristics of goat meat nuggets. The study revealed a proportional, though insignificant, increase in moisture content with higher levels of enoki mushroom stem by-products, which was accompanied by a reduction in fat percentage in the products.

#### 5.3.3. The Influence of Fruit-Derived Fibres

From the fruit class, attempts have been made to enrich meat products, specifically through the use of industry by-products to enhance their value and enhance the quality of processed meats. By-products such as apple purée [94], apple pomace [165], grape seeds [166], date seeds [167], dragon fruit peels [66,80,148], albedo, and citrus peels have been recognised as valuable sources of fibre [125,168].

Orange peel powder in different concentrations (2.5%, 5%, 7.5%, and 10%) added to beef burger formulation significantly reduced the moisture loss as the addition level increased. The ash content did not change significantly when orange peel was added in different concentrations, but it recorded a decrease directly proportional to the applied addition (from 2.91% for the control sample to 2.24% for the formulation with 10% orange peel powder) [125].

In contrast, when introducing mosambi peel powder (2%, 4%, 6%, 8%) into buffalo meat sausages and meatballs to improve their functional and physicochemical properties, as well as their fibre content, Younis et al. [168] reported an increase in moisture content, attributed to the higher water-holding capacity of the used powder. The protein content in both sausages and meatballs significantly decreased at each level of incorporation of mosambi peel powder, possibly due to the reduction in the amount of lean meat in the formulation. The fat content in both sausages and meatballs increased significantly with the incorporation of mosambi peel powder, as did the total fibre content (from 0.46% in the control sausage sample and 0.41% in the control meatball sample, to 7.33%, respectively, 6.42% in the samples with 8% powder addition). The authors attribute this effect to the high fibre content in the mosambi peel powder, which has a greater oil-binding capacity and retains fats during cooking process.

Apple puree, the main by-product of apple juice production, is an excellent source of dietary fibre and phenols. To improve the quality of dry Italian salami, Grispoldi et al. [94] introduced apple puree powder into the recipe in two variants, 7% and 14%, monitoring the behaviour of the products during the maturation period. The authors highlighted that the levels of fat and protein content decreased with the increase in the percentage of apple powder. The carbohydrate content (both simple and complex) increased from 0.9% in the control sample (without powder addition) to 8.4% (in the sample with 14% apple powder), an increase explained by the content of complex carbohydrates and, in particular, fibre in the apple puree. Similar results were reported by Younis and Ahmad [165] with the addition of the same by-product in buffalo meat sausages. With the addition of 6% apple puree powder, an increase of over seven times the total fibre content was reported, from 0.5% in the control sample to 3.82% in the fortified sample.

Grape seed flour is also a by-product that can be used to improve the technological properties of meat products. Kurt [166] studied the effects of grape seed flour (0.75%, 1.5%, and 3.0%) on the quality of fermented dry Turkish sausages (Sucuk) during heat treatment and refrigeration storage. The addition of grape seed flour reduced the moisture values of the sausages on the 7th and 14th days of the maturation period, with the level of addition having a significant influence only on the 7th day of maturation. In addition, the percentage of grape seed flour did not show significant differences in terms of fat and protein content in the sausages. However, the fat and protein values increased significantly during the maturation period due to the recorded moisture losses.

Bananas are among the most popular fruits, ranking at the top of the most produced, marketed, and consumed fruits worldwide [169]. However, approximately 20% of this production is discarded due to size and appearance defects [68]. Bananas, especially green bananas, have a valuable nutritional profile, being rich in indigestible carbohydrates such as dietary fibres and resistant starch, antioxidants, polyphenols, essential minerals like potassium, and vitamins such as provitamin A, carotenoids, B1, B2, and C [69,170]. Moreover, by-products of banana production, such as pseudo stems [98], peels [91], and flowers [171], have also been studied as potential ingredients in meat products. Alves et al. [68] studied the production of healthier bologna-type sausages using pork skin and green banana flour as fat replacers. The authors replaced back fat in the sausage recipe with a gel obtained from pig skin and green banana flour in five quantitative varieties: 20%, 40%, 60%, 80%, and 100%. The effect of replacing pig back fat with this gel on the physicochemical properties of bologna-type sausages showed a significant increase in moisture content, directly proportional to the replacement percentage, along with a decrease in fat content.

In another study, Salazar et al. [69] used whole banana flour, banana pulp flour, and banana peel flour as fibre sources in frankfurter sausage formulations. The formulations with whole banana flour had the same water content as the control formulation, both showing lower moisture compared to the variant that contained banana pulp flour. The formulation with banana peel flour exhibited a higher water content, which can be attributed to the significantly higher fibre content. The fibre and ash contents of the sausages influenced by the corresponding contents present in the added ingredients. Due to the higher ash content (~6%), banana peel flours determined a higher ash content in the sausages. Similarly, the sausages with banana peel flour exhibited the highest fibre level of 7.30%. This fibre content allows the labelling of the sausages as “high in fibre” products, considering that a product can be classified as a “source of fibre” only if it contains a minimum of 3 g of fibre per 100 g (or 1.5 g of fibre per 100 kcal), and “high in fibre” if the product contains a minimum of 6 g of fibre per 100 g (or 3 g of fibre per 100 kcal) [127].

#### 5.3.4. The Impact of Oilseed-Derived Fibres

Oilseeds and nuts have gained growing attention as functional ingredients for enriching meat products due to their high fibre and fatty acid content [172]. In this context, Basiri et al. [102] reported that incorporating linseed powder (3%, 4%, and 5%) led to an increase in fat content of raw burgers, attributed to the naturally high-fat content of flaxseeds (41.2%). Furthermore, the cooking method, specifically frying, resulted in a further increase in fat content across all beef burger formulations. In terms of moisture content, all treatments exhibited lower values than the control group prior to frying. However, after frying, the moisture content decreased significantly in all formulations.

Reddy et al. [73] obtained similar findings when incorporating flaxseed flour at 2%, 4%, and 6% into restructured buffalo meat slices. The addition significantly increased fat content in raw and cooked products, attributed to the naturally high-fat content of flaxseed flour (37.67%). Furthermore, moisture content in all three formulations was higher than in the control sample, both before and after thermal treatment. This effect was likely due to the formation of a protein network with the mucilage from flax seeds, which enhanced water retention during thermal processing.

In the study on the effect of enrichment with hemp seeds, flour, and oil on the chemical composition of a poultry roast, Augustyńska-Prejsnar et al. [145] reported a significant increase in fibre content compared to the control sample, especially for the variant with 8% added hemp seeds. This variant also exhibited the highest protein level and the lowest lipid content, explained by the lower percentage (2%) of added flaxseed oil.

### 5.4. The Effect on Sensory Characteristics and Consumer Perception

Sensory perception during food consumption arises from stimuli generated by interactions between sensory organs and the product itself. These stimuli represent the pathway through which information reaches the brain, via the central nervous system, where it is interpreted by specific receptors in the form of a perception. The formed sensory perception is translated into an individual response, measured objectively by the intensity of the stimulus and subjectively by statements people make about the perceived sensations [173].

In most cases, when a classic product is modified by introducing an element of innovation, the changes in composition also induce changes in sensory perception of consumers. Although sensory changes are inevitable, optimising recipes and processing techniques is essential to minimise the negative impact on the sensory attributes of the final product. The major changes that occur in meat products upon the incorporation of dietary fibres target colour, taste, smell, and texture thus producing a specific response from consumers and influence their acceptability of the products [103].

In general, the type of fibre and the level introduced are the factors that determine the magnitude of these sensory changes. Argel et al. [126] conducted a sensory test on four burger formulations in which a certain amount of meat (150 g·kg^−1^) was replaced with legume flour (peas, chickpeas, lentils, and beans). The sensory differences regarding appearance, taste, texture, and overall acceptability compared to a commercial sample were noticeable, but not significant. Thus, out of the group of 60 evaluators, 41.6% appreciated the taste, 61.1% the texture, 30.5% the colour, 36.1% the appearance, and over 44.4% agreed with the overall acceptability of all burger formulations.

In the sensory evaluation of turkey pâté varieties enriched with plant-based supplements (rice, oats, corn, and buckwheat), Kambarova et al. [89] reported that panellists highlighted positive changes in terms of aroma, juiciness, and overall acceptability. The visual evaluation of all pâté formulations did not reveal any apparent defects. All samples exhibited a finely divided structure, moderately uniform, with a clean and dry surface.

The sensory evaluation of chicken sausage varieties with added sugarcane fibre suggests that incorporating up to 3% sugarcane fibre does not negatively affect the overall sensory properties [90]. In the panellists’ perception, the applied addition did not significantly influence the appearance, probably due to its similar colour to chicken meat. In contrast, in the case of the association of a higher level of sugarcane fibre (3% and above) with a high-water content (15%), the appearance scores were lower, due to the dilution of the meat specific red pigment and the paler colour of the products.

Another study evaluated the sensory changes in chicken sausages enriched with banana peel powder (2%, 4%, and 6%) [91]. The sensory panel described that a 2% level of banana peel powder significantly improved hardness compared to the control group and the other treatments. However, taste, juiciness, colour, and overall acceptability did not show significant differences between the 2% banana peel powder and the control group. Although the product with 2% banana peel powder showed a high acceptability due to the water retention properties of the carbohydrates in the banana peel powder, improving juiciness and tenderness, the study suggests that adding banana peel powder at higher levels (4–6%) significantly reduced flavour and appearance of the treated sausages. Moreover, banana peel powder levels exceeding 4% resulted in a firmer texture, negatively affecting overall acceptability.

The sensory profile of meatballs with added rye bran and pea fibres showed that the main variation in the perception of sensory attributes was dose-dependent [174]. Therefore, increasing the dose of rye bran led to changes in the internal pigment, a grainy aroma and taste, and a more granular texture. Regarding pea fibre, as the addition level increased, the main difference was noted in texture; the meatballs became more granular, crumbly, firmer, and less juicy, while the aroma and taste were only partially influenced. Regarding consumer perception, a consumer appetite study showed that they appreciate meatballs with up to 5.5 g of fibre from rye bran, despite the more pronounced cereal smell, taste, and texture. The increased granular and crumbly texture, associated with higher levels of pea fibre (5.5% and above), negatively affects consumer preference in the appetite study [174].

Similarly, Momchilova et al. [156] reported texture as the determining factor in the evaluation of sausages with added inulin gel and oat bran flour. Consumers found the sausages prepared with inulin gel (as a substitute for animal fat) less attractive compared to the traditional ones, due to a softer consistency, described as “atypical” for this type of product. Additionally, the flavour of these products was described as milder compared to the commercial version.

Boișteanu et al. [175] studied how modifying a classic, well-known product (chicken roulades) by introducing a plant ingredient (spinach filling) affects consumer perception and acceptability. The sensory tests applied by the authors highlighted that the chicken roulade varieties with spinach filling received a positive evaluation compared to the traditional without filling. The hedonic test, a primary indicator of consumer perception, showed higher mean scores regarding attributes such as appearance, smell, taste, and texture for the spinach-stuffed roulade varieties. The results of check-all-that-apply (CATA) test characterised this product with positive attributes such as savoury flavour, overall pleasant appearance, characteristic meat aroma, uniform colour on the surface, uniform filling colour, balanced meat/filling taste, and balanced filling flavour intensity. The authors concluded that there is consumer acceptability for meat products with vegetable additives, which means an opportunity for the meat industry to grow in innovation and competitiveness.

Regarding the general consumer perception of fibre-enriched meat products, a recent study conducted in Slovakia revealed that over 30% of consumers regularly consume functional fibre-enriched low-calorie meat products (more than three times a week—1.4%; two–three times a week—4.7%; once a week—15.1% and once a month—22.6%), their motivation for purchasing this type of products were primarily linked to health benefits [176]. Moreover, Curutchet et al. [177] state that consumer acceptance of fibre-enriched meat products is correlated with the level of information provided. For instance, the authors demonstrated that burgers formulated with brewer’s spent grain were better received when there was strategic communication informing the consumers about fibre enrichment.

### 5.5. Functional Role of Dietary Fibres in Meat Product Stability

The integration of dietary fibres into meet products not only impacts their nutritional quality, improves the technological stability of the finished product, enhances texture and moisture retention, but also significantly contributes to maintaining quality characteristics during storage, primarily by inhibiting lipid oxidation and slowing microbial proliferation. In a study that incorporated apple peel and kinnow rind powder into restructured buffalo meat fillets, Ahmad et al. [178] demonstrated enhanced product stability under aerobic refrigerated storage, lower lipid oxidation, delayed microbial growth, and improved moisture retention, resulting in better preservation of the products. Zargar et al. [179] demonstrated that incorporating 12% pumpkin pulp into chicken sausages enhanced product stability by lowering lipid oxidation (as measured by TBARS values). Despite the slight increase in microbial counts over time, the authors state that treated sausages remained safe and of good quality, benefiting from pumpkin’s natural antioxidants and fibre content. A significant reduction in lipid oxidation, indicated by lower TBARS values, was also reported in frankfurter sausages enriched with citrus fibre. The antioxidant effect was attributed to the flavonoid content of citrus fibre, which contributed to improved oxidative stability [180]

In another study, Mushtruk et al. [181] tested the possibility of incorporating up to 5% wheat fibre with pumpkin pectin in cooked sausages. Microbiological studies were carried out on finished products immediately after preparation, and after 4, 8, 10, 12, and 15 days of storage, confirming good organoleptic quality and microbiological stability over the 15-day refrigerated storage period. Although lipid oxidation was not quantified, the absence of off-flavours or rancidity suggests oxidative stability at the inclusion level established and studied by the authors. These studies support the multifunctional role of dietary fibres as not only nutritional enhancers but also technological agents that extend shelf life and maintain the quality during storage.

## 6. Conclusions

The need to innovate meat products by adding plant-based ingredients, the main sources of fibre, arose from the necessity of incorporating this essential element into the diet, for the proper functioning of the human organism. Due to fibre deficiency in meat, and implicitly in processed meat products, this fortification can contribute to the nutritional improvement of these increasingly consumed products (due to current consumption trends of diets rich in protein foods, mainly animal products).

In general, the incorporation of dietary fibres is favoured by the minced structure of the product. Thus, the majority of experimental studies present this element incorporated into minced meat in various forms (products such as salami, sausages, burgers, meatballs, or emulsified products with a higher degree of grinding) and restructured products (such as meatloaf, restructured meat slices, roulades). The selection and use of various types of fibres can be adapted according to the specific characteristics and requirements of each product, in order to achieve optimised results in terms of physicochemical, sensory, and nutritional properties.

Therefore, choosing the type of additive, the level added, and the category of the product to be obtained is of utmost importance in achieving positive results. These aspects must be taken into consideration because the fibres from various plant sources exhibit distinct functional properties (gel formation capacity, solubility, viscosity, water binding capacity, and oil absorption) that directly influence the entire manufacturing process, as well as the characteristics of the final product.

The main changes that occur in the product enriched with dietary fibres refer to technological, physicochemical, nutritional, and sensory properties. Due to the specific functional properties of fibres, some benefits to the meat products refer to improving water retention capacity, stabilising the emulsion (specific to emulsified products), reducing cooking losses, and implicitly increasing cooking yield. These effects trigger a chain of other changes, such as maintaining juiciness due to lower losses during thermal treatment and preserving aroma and flavour through the slower release of volatile compounds, due to the fibres’ ability to retain water.

In addition to improving technological qualities, fibres can influence the chemical and physical properties of meat products by reducing fat content, increasing fibre content, enhancing texture and juiciness, and modifying taste, aroma, and colour. Therefore, the observations reveal that enriched products may have a lower energy value due to a reduced fat content, while the fibre content and other essential nutrients specific to the added ingredient are increased. These aspects are extremely important in the context of promoting a healthy and balanced diet.

The sensory changes that occur when incorporating dietary fibres into meat products can be a challenge, but not an obstacle. Through optimisation it is possible to obtain products with an improved nutritional profile and high sensory acceptability, satisfying consumer demand for healthier products.

In the strategies for optimising meat products through the addition of non-meat ingredients, the emphasis is placed on the correct choice of fibre type, mainly those with fine granulation and neutral taste, on conducting trials to determine an optimal inclusion level, and on balancing the nutritional benefits with the sensory acceptability of the final product. The production of meat products fortified with fibres from plant sources is an innovative solution that combines several benefits related to the nutritional improvement, the regulation of fibre intake, meeting the demand of consumers who emphasise a healthy lifestyle, as well as hybrid foods specific to a busy lifestyle. Therefore, given the well-recognised health benefits of dietary fibres, a future research direction should explore the potential of fibre-enriched meat products on the long-term health effects, such as supporting cardiovascular and gastrointestinal health, over extended periods of consumption.

## Figures and Tables

**Table 1:** Reported proximate composition of meat cuts from different species.

Species	Cut	Chemical Composition (%)					Ref.
		Moisture	Dry Matter	Protein	Lipids	Ash	
Beef	Loin		36.28 ± 1.42	19.30	8.03	0.35	[28]
		48.92 ± 3.42		15.69 ± 1.04		2.58 ± 0.41	[29]
	Tenderloin		41.18 ± 2.51	16.48	9.17	0.29	[28]
	Top loin	59.4		27.9	11.2	1.03	[30]
	Sirloin	67.13		21.18	9.90		[31]
	Longissimus dorsi	67.7	32.3	19.77	9.87	1.67	[32]
	Longissimus thoracis et lumborum	73.49		24.02	1.40	1.06	[33]
	Rib		35.97 ± 1.51	16.88	8.06	0.24	[28]
		45.39 ± 3.41		14.75 ± 1.09		3.02 ± 0.48	[29]
	Chuck	53.27 ± 2.94		16.78 ± 0.82		2.82 ± 0.74	[29]
	Round	53.99 ± 2.63		17.58 ± 0.71		3.10 ± 0.36	[29]
	Rump	69.85		21.67	6.59		[31]
	Butt	70.71		21.46	5.99		[31]
	Brisket		30.00 ± 1.01	19.40	5.97	0.38	[28]
	Shank		29.00 ± 1.36	18.88	5.57	0.39	[28]
Pork	Loin chop	74.1 ± 0.7		23.2 ± 0.8	1.75 ± 0.21	1.22 ± 0.04	[34]
	Loin	73.5		21.1	3.9	1.14	[35]
		67.38 ± 0.63		21.15 ± 0.38	10.60 ± 0.95	0.95 ± 0.03	[24]
	Shoulder	73.3		18.0	7.5	1.02	[35]
		72.02 ± 0.38		19.35 ± 0.26	7.52 ± 0.50	0.94 ± 0.01	[24]
	Leg	74.4		19.8	4.3	1.12	[35]
	Hind leg	70.28 ± 0.42		20.48 ± 0.22	8.33 ± 0.48	0.93 ± 0.01	[24]
Sheep	Shoulder		25.689	21.682	11.694 *	0.963	[36]
	Rack		25.989	22.499	10.694 *	0.924	[36]
	Loin		26.750	22.526	10.718 *	0.992	[36]
	Leg		26.917	21.953	10.553 *	0.929	[36]
Lamb	Loin			22.9	4.5		[37]
	Loin + rack	66.2		13.3	19.0	0.90	[38]
	Eye of shoulder			17.1	15.3		[37]
	Shoulder-ribs	68.2		16.1	14.6	1.06	[38]
	Shoulder	70.3		16.1	12.7	0.95	[38]
	Knuckle			20.5	6.8		[37]
	Leg	71.7		15.8	11.5	0.94	[38]
	Topside			22.5	6.6		[37]
Goat	Loin	75.00		21.60	1.48	1.41	[39]
	Leg		23.12	20.19	2.18	4.79	[40]
	Rump	75.49		21.30	1.40	1.25	[39]
	Shoulder		22.61	21.36	3.95	4.70	[40]
	Chest		24.26	20.20	2.29	4.98	[40]
Chicken	Breast	74.1		22.8	0.87	1.37	[41]
		74.71		23.04	1.48	0.86	[42]
	Thigh	74.50		19.02	3.58	0.76	[42]

*: mean values for meat from sheep slaughtered at different ages (1, 2 and 3 years).

**Table 2:** Use of dietary fibres in different meat product formulations.

Meat Product Formulation	Dietary Fibre	Levels Used	Objective for Use	Ref.
Goat meat nuggets	Moringa pod powder	1.5% and 3.0% (*w*/*w*)	Antioxidant capacities	[25]
	Enoki mushroom (*Flammulina velutipes*) stem wastes	2.0%, 4.0%, and 6.0% (*w*/*w*)	Value-added functional ingredient	[77]
Chicken meat nuggets	Lotus (*Nelumbo nucifera*) root powder	1.5%, 3.0%, and 4.5% (*w*/*w*)	Fibre fortification	[78]
	Rice bran	5.0% and 10% (*w*/*w*)	Increase the fibre content of meat products	[79]
Fish nuggets	Dragon fruit peel powder	1.0%, 1.5%, and 2.0% (*w*/*w*)	Quality and shelf-life improvement	[80]
Emulsion-type pork sausages	Oat bran powder	3.0%, 6.0%, and 9.0% (*w*/*w*)	Improving physico-chemical quality	[81]
Emulsion-type chicken sausage	Orange fibre, wheat fibre, bamboo fibre, carrot fibre	1.0% (*w*/*w*)	Quality maintenance during storage	[82]
Frankfurters	Cacao pod husk flour	1.5% and 3.0% (*w*/*w*)	As starch replacement for reformulated frankfurters	[61]
	Seaweed dietary fibre	2.5%, 5.0%, 7.5%, 10%, and 12.5% (*w*/*w*)	Phosphates alternative	[83]
Frankfurter-type sausage	Buckwheat husk	1.0%, 2.0%, and 3.0% (*w*/*w*)	Source of bioactive compounds	[84]
Emulsified meat products	Chia (*Salvia hispanica* L.) mucilage powder	2.5% and 5.0% (*w*/*w*)	Fat substituent	[85]
Bologna pork sausages	Beet powder	0.1%, 0.3%, and 0.5% (*w*/*w*)	Nitrite substitute	[86]
Bologna chicken sausages	Inulin, oat fibre, psyllium	3.0% and 6.0% (*w*/*w*)	Reducing fat content	[87]
Mortadella	Orange fibre, wheat fibre, bamboo fibre, carrot fibre	1.0% (*w*/*w*)	Development of functional meat products	[88]
Turkey pâté	Grain mix supplement (with oat, rice, corn, and buckwheat)	18.0% (*w*/*w*)	Protein-herbal supplement	[89]
Chicken sausage	Sugarcane fibre	2.0% and 3.0% (*w*/*w*)	Improving functional and eating qualities	[90]
	Banana peel powder	2.0%, 4.0%, and 6.0% (dry matter)	Enriching meat products with fibres	[91]
Sausage meat	Freeze-dried vegetables	1.0%, 1.6%, 2.2%, 2.8%, 3.4%, and 4.0% (*w*/*w*)	Phosphate substitutes	[92]
Liver sausages	Walnut paste	5.0%, 10.0%, 15.0%, 20.0%, and 25.0% (*w*/*w*)	Nutritional enrichment and functional value	[93]
Italian salami	Apple pomace (rehydrated, from dried powder)	7.0% and 14.0% (*w*/*w*)	Enhancing nutritional and antioxidant properties	[94]
Italian type salami	Inulin, fructo-oligosaccharide, α-cyclodextrin	2.0% (*w*/*w*)	Pork fat substitute	[95]
Canned meat products	Preparations of barley, wheat and oat fibre	3.0% and 6.0% (*w*/*w*)	Colour improvement	[96]
Comminuted meat products	Inulin, chitosan, carboxymethyl cellulose, pectin, cellulose	2.0% (*w*/*w*)	Fibre enrichment/fat substitutes	[62]
Pork meatballs	Kiwi fruit pomace dietary fibre	0.5%, 1.0%, 3.0%, 5.0%, and 7.0% (*w*/*w*)	Fat substitute	[97]
Minced pork batter	Banana pseudo-stem powder	1.5, 3.0, and 4.5 (g/kg)	Improve oxidative stability	[98]
Turkey meat batters	Citrus, apple, pea, bamboo, sugar cane fibre	2.0% (*w*/*w*)	Texture and microstructure improvement	[99]
Beef loaves	Apple, oat, pea, inulin	6.0% (*w*/*w*)	Improving the textural and nutritional properties	[74]
Buffalo meat slices	Flaxseed flour	2.0%, 4.0%, and 6.0% (*w*/*w*)	Binding properties	[73]
Beef patties	*Moringa oleifera* leaves extract powder	1.0%, 2.0%, 3.0%, and 4.0% (*w*/*w*)	Minimise shrinkage during well-done cooking methods	[100]
Beef burger	Red lentil	5.0% and 10.0% (*w*/*w*)	Quality improvement and fat reduction	[101]
	Brown linseed powder	3.0%, 4.0%, and 5.0% (*w*/*w*)	Quality attributes improvement	[102]
	Guava and tomato waste powders (peels and seeds)	5.0%, 10.0%, and 15.0% (*w*/*w*)	Enhance and improve shelf-life	[76]
Hybrid chicken meat burger	Pulse flours: yellow pea, chickpea, lentil	25%, 50%, and 75% (*w*/*w* meat replacement)	Sustainable protein source	[103]

**Table 3:** Sources of dietary fibres and their use in meat products.

Principal Groupings	Dietary Fibre Components	Fibre Sources	Specific Ingredient Added	Meat Product	Technological Effect in the Meat Product	Ref.
Non starch polysaccharides and oligosaccharides	Cellulose, hemicellulose	Cereal grains and bran	Oat bran	Rabbit meat burgers	Increased water-holding capacity, cooking yield, and firmness; reduced moisture content.	[120]
			Rice bran	Meatballs	Reduced total fat and trans fatty acids; increased firmness and reduced chewiness.	[121]
		Herbaceous plant	Sugarcane fibre	Chicken sausages	Improved cooking yield; higher phenolic content and antioxidant activity; reduced TBARS values.	[90]
		Cereal and legume flours	Soybean, cowpea and groundnut	Chicken nuggets	Reduced moisture content; improved texture, binding ability, and firmness; increased cooking yield.	[122]
	Cellulose, hemicellulose, pectin	Fruits and vegetables	Moringa pod powder	Goat nuggets	Improved emulsion stability, cooking yield, and moisture retention; reduced expressible water and hardness.	[25]
			Dragon fruit peel powder	Alpaca sausages	Increased water retention and activity; reduced fat content and chewiness.	[66]
	Inulin, fructo-oligosaccharides	Chicory root, jerusalem artichoke (inulin extracted from simple carbohydrates)	Emulsion gel (with 16.5% inulin)	Bologna sausages	Improved heat stability; reduced hardness.	[123]
			Jerusalem artichoke powder	Sausages	Reduced TBARS values; increased moisture and adhesiveness; reduced hardness.	[124]
	Gums and mucilages, psyllium	*Plantago ovata* plant	Psyllium powder	Chicken bologna sausages	Improved adhesiveness and hardness; reduced gumminess and cohesiveness.	[87]
	Gums and mucilages	Oilseeds	Flaxseed flour	Restructured buffalo meat fillets	Formed a gel network with increased cohesiveness and reduced hardness, gumminess, and springiness.	[73]
		Herbaceous plant	Chia seeds	Meat emulsion	Increased emulsion stability; improved hardness and cohesiveness.	[85]
Carbohydrate analogues	Pectin	Fruits and vegetables	Guava and tomato waste powders	Beef burgers	Reduced shrinkage and cooking loss; enhanced water-holding capacity	[76]
			Orange peel powder	Beef burgers	Improved texture and cooking yield; increased phenolic content; delayed lipid oxidation.	[125]
	Resistant starch	Legumes, unripe banana	Pulse flours (lentil, pea, chickpea, bean)	Burgers	Increased cooking yield, decreased diameter reduction and expressible liquid.	[126]
			Green banana flours (pulp, peel and whole banana)	Frankfurters	Increased moisture retention; higher cooking yield; reduced shrinkage.	[69]
Lignin	Lignin	Woody plant	Cacao pod husk flour	Frankfurters	Enhanced emulsion stability; increased hardness and reduced cohesiveness.	[61]
Non starch polysaccharides and oligosaccharides	Cellulose, hemicellulose					

**Table 4:** Comparative functional properties of selected dietary fibre sources.

Dietary Fibre Source	Water Retention Capacity (g/g)	Oil-Holding Capacity (g/g)	Gel-Forming Ability (Swelling Capacity, SWC—mL Water/g)	Ref.
Grapefruit peel	13.43	22.10	High	[130]
Lemon	1.1	9.3	High (SWC = 9.9)	[131,132]
Orange	7.1–12.6	0.86–1.28	High	[118,133]
Kiwifruit	2.02–13.34	22.79–23.00	-	[134]
Apple pomace	7.5	2.2	High (SWC = 7)	[135]
Pear pomace	3.44–4.9	1.09–2.77	Moderate (SWC = 5.9)	[135,136]
Date	5.7	2.3	Low (SWC = 3.9)	[135]
Banana peel	6.57	4.75	Low to moderate (4.3–4.8)	[137,138]
Rice bran	4.75–5.06	4.58–5.64	Low (SWC = 2.02–2.72)	[139]
Oat fibre	3.42	3.95	Moderate to high	[140]

## Data Availability

No new data were created or analysed in this study. Data sharing is not applicable to this article.
